# Care burden and psychological factors in family caregivers of diabetic patients

**DOI:** 10.1590/1806-9282.20260104

**Published:** 2026-07-31

**Authors:** Zeynep Irmak Kaya, Hatice Hamarat, Sevilay Süreyya Ermiş, Ayşe Bağcı Akçeşme, Alaettin Ünsal

**Affiliations:** 1 Eskişehir City Hospital, Department of Internal Medicine – Eskişehir, Türkiye.; 2 Eskişehir Osmangazi University, Faculty of Medicine, Department of Internal Medicine, Division of Endocrinology and Metabolism – Eskişehir, Türkiye.; 3 Eskişehir Osmangazi University, Faculty of Medicine, Department of Public Health – Eskişehir, Türkiye.

**Keywords:** Diabetes mellitus, Caregivers, Caregiver burden, Depression, Anxiety, Stress

## Abstract

**OBJECTIVE:**

The aim of this study was to evaluate the levels of caregiver burden among family members of patients with type 2 diabetes mellitus and to investigate the sociodemographic, clinical, and psychological factors associated with this burden.

**METHODS:**

This cross-sectional study included 125 caregivers of patients attending the Internal Medicine Outpatient Clinic at Eskişehir City Hospital, selected via consecutive sampling. Data were collected using a sociodemographic information form, the Zarit Caregiver Burden Index, and the Depression Anxiety Stress Scale-21. Statistical analyses included independent samples t-tests, Pearson’s correlation, and a three-step hierarchical multiple linear regression.

**RESULTS:**

Zarit Caregiver Burden Index scores were significantly higher among spousal caregivers and those caring for patients with diabetic complications (p<0.001). Caregiver burden showed moderate-to-strong positive correlations with depression (r=0.48), anxiety (r=0.42), and stress (r=0.51). Patient glycated hemoglobin levels showed a strong positive correlation with both Zarit Caregiver Burden Index (r=0.684) and depression scores (r=0.592). In the final hierarchical regression model, the presence of complications (β=0.35), higher glycated hemoglobin levels (β=0.32), spousal caregiving (β=0.28), stress (β=0.24), and depression (β=0.19) were identified as significant independent predictors of burden. The final model accounted for 51.2% of the total variance (Adjusted R^2^=0.484).

**CONCLUSION:**

Caring for individuals with diabetes mellitus is associated with a substantial psychological burden on family caregivers. These findings highlight the importance of integrating systematic psychosocial assessment and targeted support into routine diabetes management, particularly for caregivers of patients with poor glycemic control and complications, to mitigate caregiver burden and distress.

## INTRODUCTION

Diabetes mellitus (DM) is a chronic condition requiring lifelong monitoring and treatment, substantially affecting daily functioning and quality of life. Effective diabetes management extends beyond pharmacological therapy to include dietary regulation, blood glucose monitoring, prevention of complications, and lifestyle modifications. Within this context, family members or close associates frequently assume a central role in the caregiving process.

Caregiver burden is a multidimensional construct encompassing both objective and subjective components. Objective burden refers to the tangible demands and observable costs of caregiving, such as financial strain and assistance with daily activities, whereas subjective burden reflects the caregiver’s emotional and psychological response to these demands^1^. Importantly, caregiving is not exclusively associated with negative outcomes; it may also involve positive experiences, often described as “caregiving gain,” including personal growth, meaning-making, and role satisfaction^2^.

In many non-Western societies, including Turkey, caregiving is shaped by strong cultural norms rooted in familism and filial responsibility. Within this sociocultural framework, caring for an ill family member is often perceived as a moral obligation rather than an individual choice. While this perspective may reinforce family cohesion, it can also intensify both the objective and subjective dimensions of caregiver burden.

The sustained nature of caregiving exerts multifaceted effects on caregivers, including physical fatigue, emotional exhaustion, and social limitations. Evidence indicates that caregiver burden in diabetes is closely associated with reduced quality of life and increases significantly in the presence of disease-related complications^3^. Beyond physical demands, caregiver burden has been consistently linked to psychological distress, including depression, anxiety, and stress, across both type 1 and type 2 diabetes populations^4,5,6^.

Despite growing recognition of these associations, studies employing comprehensive multivariate models to simultaneously evaluate clinical and psychological determinants of caregiver burden remain limited. In Turkey, research addressing these factors within clinical populations is particularly scarce, highlighting a critical gap in the literature.

This study aimed to address this gap by examining the interplay between clinical factors (glycemic control as measured by HbA1c and diabetic complications) and psychological variables in shaping caregiver burden. Given the cultural context in which caregiving responsibilities are strongly embedded, these relationships may be particularly pronounced. We hypothesized that: (i) higher HbA1c levels and the presence of diabetic complications would be associated with increased caregiver burden; and (ii) psychological distress (depression, anxiety, and stress) would independently predict caregiver burden after adjustment for sociodemographic factors.

## METHODS

### Study design and setting

This cross-sectional study was conducted among caregivers of patients with type 2 diabetes mellitus followed up at the Internal Medicine Outpatient Clinic of Eskişehir City Hospital, Turkey. Caregivers aged ≥18 years who had provided unpaid care to a patient with type 2 diabetes for at least 6 months were included. This 6-month threshold was chosen to ensure that participants had experienced sustained caregiving demands rather than acute adjustment stress.

Inclusion criteria were: (i) age ≥18 years, (ii) providing unpaid care to a patient with type 2 diabetes, (iii) caregiving duration ≥6 months, and (iv) ability to read and understand Turkish. Exclusion criteria were: (i) being a paid professional caregiver, (ii) caring for a patient with type 1 diabetes, and (iii) having a diagnosed severe psychiatric disorder that would impede accurate self-report.

### Participants and sampling

The study population comprised caregivers of diabetic patients attending the Internal Medicine Outpatient Clinic during the study period. Consecutive sampling was used; all eligible caregivers presenting to the clinic were invited to participate.

Sample size was calculated using G Power version 3.1.9.7^7^. Based on a medium effect size (f^2^=0.15), alpha=0.05, and power=0.95 for multivariate linear regression, the minimum required sample size was 107. To account for potential data loss, 125 caregivers were enrolled.

**Figure 1:**
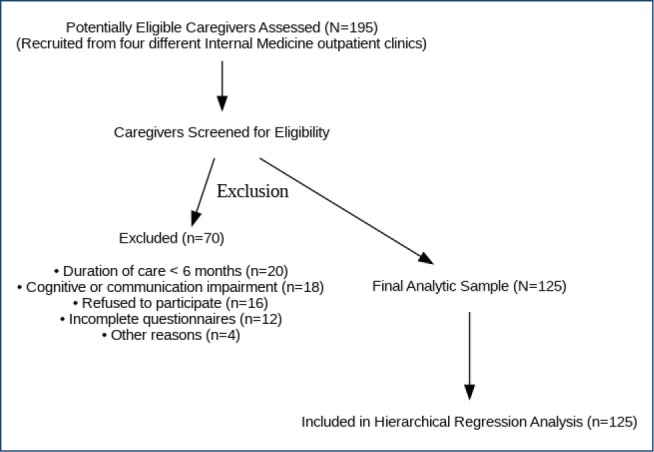
Flowchart of the study participant recruitment and selection process.

Participants were recruited sequentially from the clinics of four different specialists between June 2024 and September 2025. Of 195 potentially eligible caregivers assessed, 70 were excluded: caregiving <6 months (n=20), cognitive/communication impairments in patient or caregiver (n=18), refusal to participate (n=16), incomplete questionnaires (n=12), and other technical reasons (n=4). The final analytic sample consisted of 125 caregivers (Figure 1). This multi-practitioner, sequential recruitment strategy was designed to minimize selection bias and provide a representative cohort.

### Data collection tools

#### Sociodemographic and clinical ınformation form

The form captured sociodemographic variables (age, gender, relationship to patient, and caregiving duration) and clinical data on diabetic complications (vision loss, chronic kidney failure, diabetic foot syndrome, and neuropathy), diagnosed according to American Diabetes Association criteria^8^. The patient’s most recent HbA1c level (within 3 months) was recorded from medical records.

Glycemic control classification (descriptive only): For descriptive purposes, patients were stratified into three groups: well-controlled (HbA1c <7.5%), suboptimally controlled (7.5–9.0%), and poorly controlled (>9.0%). The 7.5% threshold aligns with American Diabetes Association (ADA) recommendations for individualized targets in older adults or those with longer disease duration or higher complication burden^8^. Importantly, HbA1c was entered as a continuous variable in all primary regression analyses.

#### Zarit Caregiver Burden Scale

Caregiver burden was assessed using the ZBI^9^, which has been validated in Turkish by İnci and Erdem^10^. The scale consists of 22 items rated on a five-point Likert scale (0=never to 4=almost always), yielding total scores from 0 to 88; higher scores indicate greater burden. In this study, internal consistency was high (Cronbach’s α=0.89).

#### Depression Anxiety Stress Scale-21

Psychological distress was assessed using the DASS-21^11^, with Turkish validity and reliability established by Yılmaz et al.^12^. Each subscale (depression, anxiety, and stress) contains seven items rated on a four-point scale. Subscale scores were multiplied by two to obtain DASS-42-equivalent scores. Clinical severity thresholds (depression ≥10, anxiety ≥8, and stress ≥15 for mild symptoms) were applied as defined in the original DASS manual^11^. The Turkish adaptation confirmed reliability but did not establish separate normative cutoffs; therefore, the original criteria were used. Internal consistency was excellent (Cronbach’s α: depression=0.86, anxiety=0.84, stress=0.88).

### Data collection procedure

After obtaining written informed consent, questionnaires were self-administered, with assistance provided to 15.2% of participants to ensure data integrity. A researcher provided standardized instructions and answered questions, but did not record responses. Completion took 15–20 min. Data collection took place from June 2024 to September 2025.

### Ethical approval

The study was approved by the Non-Interventional Clinical Research Ethics Committee of Eskişehir City Hospital (Approval No: 2024/05-12). Written informed consent was obtained from all participants. The study complied with the Declaration of Helsinki. All data were pseudonymized and stored on a secure, password-protected local server accessible only to the research team.

Data availability statement: The data supporting this study are available from the corresponding author upon reasonable request. Due to ethical and privacy considerations, the data are not publicly available.

### Statistical analysis

Hierarchical multiple linear regression was performed to identify independent predictors of caregiver burden (ZBI score). Variables were entered in three blocks:

Step 1: Demographic variables (age, gender, and relationship: spouse vs. non-spouse)

Step 2: Socioeconomic factor (income level: good vs. poor/moderate)

Step 3: Clinical and psychological factors (presence of diabetic complications, HbA1c [continuous], depression, anxiety, and stress)

Statistical significance was set at p≥0.05.

Prior to regression, model assumptions were evaluated. Multicollinearity was not observed among the independent variables (all variance inflation factor [VIF]<2.5). Normality of residuals was confirmed via the Shapiro-Wilk test (p>0.05) and Q–Q plots. Homoscedasticity was verified by plotting standardized residuals against predicted values. Residual independence was visually confirmed. Additional diagnostics (VIF, tolerance, Durbin-Watson, and Cook’s distance) are provided in the Supplementary Figure 1.

Statistical analyses were performed using IBM SPSS Statistics for Windows, Version 26.0 (IBM Corp., Armonk, NY, USA)^13^.

## RESULTS

### Sample characteristics

The study sample consisted of 125 family caregivers and their care recipients with Type 2 diabetes mellitus. The patients had a mean age of 62.4±10.8 years (range: 42–85 years), and 54.4% (n=68) were female. The mean duration of diabetes was 11.4±6.8 years, and 64.0% (n=80) of the patients were receiving insulin therapy. Additionally, 57.6% (n=72) had at least one diabetic complication (38 severe and 34 mild), while 42.4% (n=53) had no documented complications. The mean HbA1c level was 8.4±1.6% (Table 1).

**Table 1:** Clinical and demographic characteristics of patients with Type 2 diabetes mellitus (n=125).

Characteristic	Value
Age (years), mean±SD (range)	62.4±10.8 (42–85)
Female gender, n (%)	68 (54.4)
Diabetes duration (years), mean±SD	11.4±6.8
Treatment modality, n (%)	
Insulin therapy	80 (64.0)
Oral antidiabetic drugs only	45 (36.0)
HbA1c (%), mean±SD	8.4±1.6
Diabetic complications	
Severe complications (vision loss, CKD, and foot ulcer)	38 (30.4)
Mild complications (neuropathy and background retinopathy)	34 (27.2)
No complications	53 (42.4)

CKD: chronic kidney disease; SD: standard deviation; HbA1c: glycated hemoglobin. Note: Data are presented as mean±standard deviation for continuous variables and as frequency (percentage) for categorical variables.

Caregivers had a mean age of 46.8±12.3 years, and 65.6% were female. The mean duration of caregiving was 4.2±2.8 years. The scores on the ZBI and the DASS-21 showed normal distribution according to the Kolmogorov-Smirnov test (p=0.086 and p=0.072, respectively). No significant floor or ceiling effects were observed in either scale (both below 15%).

Regarding the psychological status of the caregivers, the mean scores for depression, anxiety, and stress were 8.4±4.1, 7.6±3.8, and 9.2±4.5, respectively.

### Descriptive factors affecting caregiving burden (univariate analyses)

The univariate comparisons presented in Tables 2 and 3 were considered exploratory in nature. Therefore, we did not adjust for multiple comparisons, and these findings should be interpreted as hypothesis-generating rather than confirmatory.

**Table 2:** Comparison of caregiver burden and depression scores (demographics).

Variable	Category	n (%)	ZBI score (mean±SD)	p^a^	Cohen’s d	Depression score (mean±SD)	p^b^
Gender	Female	82 (65.6)	41.2±13.5	0.012	0.49	8.4±4.1	0.012
	Male	43 (34.4)	34.8±12.4			6.8±3.8	
Education	High school or below	91 (72.8)	42.6±12.8	0.042	0.35	8.9±4.2	0.042
	University	34 (27.2)	36.4±11.5			7.1±3.6	
Income	Poor/moderate	98 (78.4)	43.8±13.2	0.005	0.62	9.2±4.4	0.005
	Good	27 (21.6)	33.2±10.8			6.4±3.1	
Relationship	Spouse	48 (38.4)	45.6±14.2	<0.001	0.78	10.2±4.5	<0.001
	Non-spouse^†^	77 (61.6)	35.2±12.1			7.4±3.9	

ZBI: Zarit Caregiver Burden Index; SD: standard deviation. Statistical analysis: p^a^ indicates the significance level for ZBI scores; p^b^ indicates the significance level for depression scores (independent samples t-test or one-way ANOVA, as appropriate).

†Non-Spouse category includes adult children, grandchildren, and extended family members or neighbors who act as the primary informal caregiver. Study population: All patients were diagnosed with Type 2 diabetes mellitus. All caregivers had been providing care for at least 6 months prior to study enrollment.

**Table 3:** Comparison of caregiver burden and anxiety and depression scores (clinical factors).

Variable	Category	n (%)	ZBI score (mean±SD)	pa	Anxiety score (mean±SD)	pb	Depression score (mean±SD)	pc
Treatment	Oral antidiabetics	45 (36.0)	32.4±11.5	**0.008**	7.4 ± 3.1	**0.010**	6.1±3.2	**0.008**
	Insulin therapy	80 (64.0)	43.1±13.8		10.2±4.4		8.8±4.5	
Complications	Severe^‡^	38 (30.4)	49.6±11.2	**<0.001**	10.6±4.1	**<0.001**	11.2±3.8	**<0.001**
	Mild	34 (27.2)	43.2±13.5		8.9±3.8		8.4±4.1	
	None	53 (42.4)	28.9±10.6		7.2±3.5		6.2±3.1	
HbA1c level	Group 1: <7.5%	42 (33.6)	29.4±11.2	**<0.001**	7.1±3.3	**<0.001**	6.1±3.2	**<0.001**
	Group 2: 7.5–9.0%	48 (38.4)	41.8±12.5		9.4±3.9		8.3±4.0	
	Group 3: >9.0%	35 (28.0)	51.2±13.6		12.1±4.5		11.4±4.7	

Note: ZBI: Zarit Caregiver Burden Index; Mean±SD: mean±standard deviation; HbA1c: glycated hemoglobin; p^a^: significance level for ZBI (independent samples t-test or ANOVA); p^b^: significance level for anxiety scores; p^c^: significance level for depression scores.

‡ Severe complications include vision loss, chronic kidney failure, and diabetic foot. All patients included in the analysis were diagnosed with Type 2 diabetes mellitus, and all caregivers had been providing care for a minimum duration of 6 months prior to the study. HbA1c categories are presented for descriptive purposes only. The 7.5% threshold reflects individualized glycemic targets recommended for older adults and patients with longer disease duration, as per ADA guidelines. HbA1c was analyzed as a continuous variable in all primary regression analyses (see Table 4). Subgroup analyses (e.g., across complication severity levels) may have been underpowered due to small sample sizes in certain categories (ns=38, 34, 53); these findings should be interpreted with caution. Bold formatting indicates statistically significant results (p<0.05).

**Table 4:** Hierarchical multiple regression analysis predicting caregiver burden (Zarit Caregiver Burden Index score).

Step	Predictors	β	95%CI	t	p	∆R2	∆F	p (for∆F)
Step 1	**Demographic baseline**					**0.138**	**6.52**	**<0.001**
	Age	-0.09	[-0.21, 0.04]	-1.28	0.201			
	Gender (female)	0.21	[0.06, 0.35]	2.42	0.017			
	Relationship (spouse)	0.28	[0.12, 0.44]	3.15	0.002			
Step 2	**Socioeconomic factors**					**0.048**	**4.32**	**0.042**
	Income (good)	-0.16	[-0.30, -0.02]	-2.05	0.042			
Step 3	**Clinical and psychological**					**0.326**	**24.67**	**<0.001**
	Complications (yes)	0.35	[0.19, 0.51]	4.12	<0.001			
	HbA1c (%)	0.32	[0.16, 0.48]	3.84	<0.001			
	Depression	0.19	[0.05, 0.33]	2.58	0.011			
	Anxiety	0.08	[-0.04, 0.20]	1.32	0.189			
	Stress	0.24	[0.09, 0.39]	2.98	0.003			

Note: β: standardized regression coefficient; CI: confidence interval; ∆R^2^: change in R^2^ at each step; ∆F: F-change statistic; HbA1c: glycated hemoglobin. Multicollinearity was assessed using the variance inflation factor (VIF) and tolerance values; all VIF values were <2.5, and tolerance values were >0.4, indicating no multicollinearity. Model assumptions, including normality and homoscedasticity of residuals, were evaluated through diagnostic plots. Final model (Step 3): Total R^2^=0.512, Adjusted R^2^=0.484, F(8, 116)=18.45, p<0.001. Bold formatting indicates statistically significant results (p<0.05).

Exploratory univariate comparisons of caregiving burden and depression scores according to the participants’ sociodemographic characteristics are presented in Table 2.

Female caregivers exhibited significantly higher ZBI scores in comparison to their male counterparts (41.2±13.5 vs. 34.8±12.4; p=0.012; Cohen’s d=0.49). Caregivers who had received a university education (mean±standard deviation [SD]=36.4±11.5) exhibited significantly lower ZBI scores in comparison to those with a high school education or below (42.6±12.8) (p=0.042; Cohen’s d=0.35). In a similar vein, caregivers who described their family income as “good” exhibited significantly lower care burden scores in comparison to those who reported their income as “moderate/poor” (33.2±10.8 vs. 43.8±13.2; p=0.005; Cohen’s d=0.62). Spouse caregivers reported significantly higher ZBI scores (45.6±14.2) compared to children or other relatives (35.2±12.1) (p<0.001; Cohen’s d=0.78). Scores indicative of depression followed a similar pattern across all demographic variables.

### Relationship between caregiving burden, clinical factors, and psychological status

The psychological status of the caregivers was evaluated using the DASS-21 subscales. The mean scores for the entire cohort were 8.38±4.08 for depression, 7.62±3.75 for anxiety, and 9.18±4.46 for stress, with an overall total DASS-21 score of 25.18±11.24. These findings indicate a notable level of psychological distress in the study population. Detailed subscale distributions and internal consistency coefficients are provided in the Supplementary Table 1.

Pearson correlation analysis revealed a strong, positive, and statistically significant relationship between HbA1c levels and caregiver burden (r=0.684, p<0.001). HbA1c was also significantly correlated with depression scores (r=0.592, p<0.001). Caregiver burden showed moderate to strong positive correlations with depression (r=0.48), anxiety (r=0.42), and stress (r=0.51) (all p<0.001). In subgroup analyses, correlations between HbA1c, psychological distress, and caregiver burden appeared to be consistently stronger among spousal caregivers compared to non-spousal caregivers (Supplementary Tables 2, 3, and 4).

**Figure 2:**
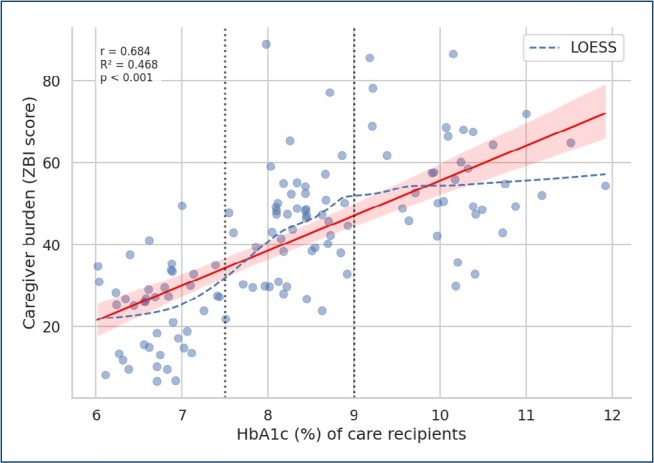
Correlation between glycemic control (HbA1c) and caregiver burden (Zarit Burden Interview scores). ZBI: Zarit Burden Index; HbA1c: glycated hemoglobin. The blue dashed line represents the LOESS (locally estimated scatterplot smoothing) curve, indicating the non-linear trend of caregiver burden. Scatterplot of patient HbA1c levels (%) versus caregiver burden (Zarit Burden Interview scores). The solid red line represents the linear regression fit, while the blue dashed line represents the LOESS (locally estimated scatterplot smoothing) curve, highlighting the non-linear trend of burden. The shaded area indicates the 95%CI. Vertical dashed lines denote the clinical HbA1c thresholds at 7.5% and 9.0%. A strong positive correlation was observed between glycated hemoglobin and Zarit Burden Index scores (r=0.684, p<0.001, n=125), indicating that poorer glycemic control is strongly correlated with significantly higher caregiver burden.

Figure 2 presents the scatterplot of patient HbA1c levels versus caregiver burden scores. Visual inspection, supported by a Locally Estimated Scatterplot Smoothing (LOESS) curve (blue dashed line), confirmed a clear positive trend between poorer glycemic control and increased burden. To verify the robustness of the observed strong correlation (r=0.684, p<0.001), a sensitivity analysis was conducted. Spearman’s rho yielded a consistent and significant positive association (rho=0.672, p<0.001), indicating that the relationship was not driven by non-normality. Furthermore, Cook’s distance analysis (Max=0.082) confirmed the absence of influential outliers, validating the stability of the regression model.

In terms of clinical significance, 67 caregivers (53.6%) had depression scores exceeding the clinical threshold (i.e., ≥10), 71 (56.8%) had anxiety scores above the threshold (i.e., ≥8), and 59 (47.2%) had stress scores equal to or exceeding the threshold (i.e., ≥15) (Supplementary Table 5). These findings suggest that a considerable proportion of caregivers experienced at least mild psychological distress. However, these prevalence estimates should be interpreted with appropriate caution. The DASS-21 has been validated in a general Turkish population (Yılmaz et al.), but normative data specific to caregiver populations are not yet available; caregivers may have systematically higher baseline distress scores compared to the general population, which could influence threshold-based prevalence estimates. Additionally, although data collection spanned June 2024 to September 2025—a period reflecting a stable post-pandemic clinical environment—residual pandemic-related distress cannot be entirely excluded as a contributing factor.

Table 3 presents exploratory comparisons based on clinical characteristics. Caregivers of patients receiving insulin therapy exhibited significantly higher ZBI scores (43.1±13.8) compared to those caring for patients on oral antidiabetics alone (32.4±11.5) (p=0.008; Cohen’s d=0.85). The presence of diabetic complications was strongly associated with increased caregiver burden; caregivers of patients with severe complications had the highest ZBI scores (49.6±11.2), followed by those with mild complications (43.2±13.5), and those without complications (28.9±10.6) (p<0.001). A similar gradient was observed for anxiety and depression scores. Detailed model diagnostics of the final regression model, including multicollinearity, residual independence, and influential observation analyses, are presented in Supplementary Table 6.

### Independent predictors of caregiving burden (hierarchical regression analysis)

Hierarchical linear regression analysis was performed to ascertain the independent predictors of caregiving burden. Multicollinearity was not observed among the included variables, as all VIF values were below 2.5. The results of the hierarchical regression analysis are presented in Table 4. As a sensitivity analysis,

Demographic variables entered in Step 1 accounted for 13.8% of the variance in caregiver burden. The addition of socioeconomic factors in Step 2 significantly increased the explained variance by 4.8% (∆R^2^=0.048, p=0.042). In the final step, clinical and psychological variables contributed an additional 32.6% to the model (∆R^2^=0.326, p<0.001). The full model explained 51.2% of the total variance in caregiver burden (R^2^=0.512, Adjusted R^2^=0.484, p<0.001).

In the final model, the presence of diabetic complications (β=0.35, p<0.001), higher HbA1c levels (β=0.32, p<0.001), spousal caregiving (β=0.28, p=0.002), and stress scores (β=0.24, p=0.003) emerged as the strongest significant independent predictors. Depression was also a significant predictor (β=0.19, p=0.011), whereas anxiety was not statistically significant (β=0.08, p=0.189). After controlling for clinical and psychological factors, gender and income level lost their statistical significance.

## DISCUSSION

The present study found that the burden on family members caring for individuals with diabetes is significant and is closely associated with both patient-related clinical characteristics and the psychological status of the caregivers. One of the most striking findings of this study is the strong association between HbA1c and caregiver burden (r=0.684, p<0.001), highlighting the close interdependence between metabolic control and caregiver well-being. This correlation suggests that as glycemic control worsens, the perceived burden on the family caregiver intensifies proportionally. This finding is particularly notable given that the strength of this association exceeds those reported in several previous studies, potentially due to the cultural expectations of caregiving in the Turkish context or the high prevalence of complications in our cohort (30.4% with severe complications). The elevated caregiver burden may be linked to difficulties in the caregiver caregiver well-being. This correlation suggests that management. Adelman et al.^14^ also identified complications as one of the strongest determinants of caregiver burden in chronic diseases. A number of studies have indicated that the burden experienced by caregivers is more closely associated with patients’ functional status and comorbidities than with a diagnosis of diabetes itself, particularly in elderly populations^15^. It is important to note that this relationship may be bidirectional; a high level of caregiver burden is associated with suboptimal dietary and medication management by the patient, which could potentially accelerate the progression of complications^14^. The findings of this study suggest that the occurrence of serious complications may transform caregiving from basic support into intensive physical and emotional labor, which may be associated with the transformation of spousal relationships into caregiver–patient dynamics^14,16^.

The strong positive correlation observed between HbA1c levels and caregiver burden (r=0.684) is a noteworthy finding. Higher HbA1c levels are indicative of suboptimal metabolic control, which frequently necessitates more intensive caregiving efforts, including frequent blood glucose monitoring, insulin adjustments, and dietary supervision. However, in accordance with Pearlin’s stress-process model of caregiving, this relationship may be indicative of a bidirectional association rather than a direct causal pathway: elevated caregiver burden may impede the caregiver’s ability to assist the patient in managing their condition, which may result in diminished glycemic control^17^. Conversely, suboptimal glycaemic control may, in turn, be associated with an augmentation in care demands and an exacerbation of the caregiver’s burden^14^. This cyclical dynamic emphasises the necessity for integrated interventions that address both patient glycaemic outcomes and caregiver well-being concurrently.

The results of the study indicated that educational level and economic status emerged as significant sociodemographic factors. Caregivers with a higher level of education reported a lower burden, consistent with the findings of Kristianingrum et al.^18^, suggesting that health literacy improves disease management skills. Conversely, low income was identified as an independent factor that exacerbated the burden, primarily due to the costs associated with medications and special nutritional requirements^5^. A qualitative study by Beverly and Wray^19^ suggested that diabetes education could alleviate psychological distress without necessarily reducing the perceived burden, implying that burden and distress may represent distinct constructs. Conversely, the present study revealed that both burden and psychological distress levels were elevated. This discrepancy may be partly explained by the high prevalence of severe complications in the present cohort (30.4%), which may have overshadowed the potential buffering effects of education alone. This suggests that the protective role of education may be overwhelmed in the context of advanced disease severity.

The high prevalence of clinically significant psychological distress in this cohort highlights the substantial mental health burden on family caregivers of patients with Type 2 diabetes mellitus. These elevated rates appear to be primarily associated with the chronic and progressive nature of Type 2 diabetes and its complications, as evidenced by HbA1c being a significant independent predictor of caregiver burden in the multiple regression analysis.

Within the sociocultural milieu of Turkey, caregiving is frequently regarded as an unavoidable familial responsibility, firmly entrenched in the tenets of filial piety and collectivist family structures. This cultural norm, when combined with traditional gender roles in which female family members typically shoulder the primary responsibility, may intensify the perceived burden^10,12^. Although not directly measured in this study, future research should examine whether caregiving satisfaction, such as moral fulfilment or strengthened family bonds, might partially mitigate psychological distress among Turkish caregivers^20^. It is therefore essential to consider this potential cultural duality when developing interventions that both address caregiver strain and acknowledge the adaptive aspects of caregiving.

Hierarchical regression analysis (Table 4) revealed that the strongest independent predictors of caregiver burden were the presence of diabetic complications, being a spousal caregiver, and higher depression and stress levels (Total R^2^=0.512). Spouse caregivers frequently report a heightened burden, a phenomenon that may be related to their advanced age and the potential presence of chronic conditions^14,16^. Within the Turkish context, in which female spouses typically assume the majority of caregiving responsibilities, this burden may be further compounded by traditional gender expectations^10,12^. Correlations appeared to be stronger among spousal caregivers (Supplementary Table 2), suggesting a potentially greater vulnerability in this group.

The moderate to strong positive correlations observed between care burden and psychological status are consistent with the findings of Bastawrous^21^ and Schulz and Sherwood^15^. Beyond the statistical correlations observed, the elevated DASS-21 scores frequently reached clinical significance thresholds in this study. This finding highlights that the distress reflects a formal need for urgent psychosocial interventions rather than merely subclinical strain.

Although anxiety was included in the regression model, it did not reach statistical significance (β=0.08, p=0.189). However, a strong association was demonstrated with complications in univariate analyses (Cohen’s d=0.85). This finding suggests that caregivers of patients with severe complications experience clinically significant anxiety symptoms that warrant attention.

### Limitations

It is imperative to acknowledge the limitations of the present study. A primary limitation is the exclusive focus on caregivers of patients with Type 2 diabetes mellitus; therefore, findings may not be generalizable to caregivers of individuals with Type 1 diabetes, whose care demands differ significantly^4,6^. The cross-sectional design precludes causal inferences regarding the direction of the observed relationships, although bidirectional pathways (e.g., caregiver burden contributing to poor glycaemic control or patient complications increasing burden) remain plausible and warrant longitudinal investigation.

Several potentially important confounders were not measured, including caregiver health status and comorbidities—which have been identified as critical confounders of caregiver burden^15^, as caregivers with poorer health may report higher burden independently of the patient’s clinical status—formal and informal social support networks, objective caregiving intensity (e.g., hours of care per week), caregiver employment status and work-related disruptions attributable to caregiving responsibilities, and formal diabetes education received by caregivers. The absence of these variables limits our ability to adjust for their potential confounding effects, and the observed associations between patient clinical factors and caregiver burden may be partially confounded by unmeasured caregiver characteristics. Additionally, the “Adult Child/Other Family Member” category combined adult children, grandchildren, extended family members, and neighbors into a single group. These subgroups may differ in caregiving intensity and relationship dynamics, but the sample size precluded separate analysis.

Another limitation is the absence of objective indicators of caregiver burden, such as the exact number of daily or weekly caregiving hours. While we captured subjective burden through the ZBI, the lack of data on caregiving duration per day prevents a more nuanced analysis of the relationship between time investment and psychological distress. Future studies should include time-use diaries to better quantify the objective workload of diabetes caregiving.

Furthermore, the multiple univariate comparisons presented in Tables 1 and 2 were performed without adjustment for Type I error (e.g., Bonferroni correction); given the exploratory nature of these subgroup analyses, emphasis should be placed on the primary hierarchical regression results (Table 4), which are less susceptible to this limitation. Despite the satisfactory overall sample size for the primary regression analysis (power=0.95), subgroup comparisons (e.g., across complication severity levels) may have been underpowered, and these findings should be interpreted with caution.

Additionally, the absence of other objective metabolic indicators and detailed functional status assessments of patients precluded further insight into the determinants of caregiver burden. Finally, the use of consecutive sampling from a single tertiary outpatient clinic may introduce selection bias, as it excludes caregivers of homebound patients with severe disability; thus, our findings may primarily represent caregivers of patients with active clinical follow-up and may not be fully generalizable to all caregiver populations. Furthermore, the “severe complications” category combined clinically heterogeneous conditions—vision loss, chronic kidney failure, and diabetic foot syndrome—which differ substantially in their caregiving demands and daily care requirements. This grouping may have obscured differential associations between specific complication types and caregiver burden; future studies with larger samples should examine each complication type separately. Additionally, the relatively high proportion of explained variance (R^2^=0.512) should be interpreted with caution, as this value may partly reflect common method variance given that all psychological measures (DASS-21) and the outcome variable (ZBI) were self-reported by the same caregiver at the same time point. Future studies incorporating multi-informant designs or objective burden indicators would help clarify whether this level of explained variance reflects true predictive strength or methodological artifact.

## CONCLUSION

The present study demonstrates that the burden experienced by caregivers of individuals with Type 2 diabetes mellitus is significant, and that this burden is closely associated with diabetic complications, being a spousal caregiver, and psychological distress including depression, anxiety, and stress. The strong correlation between HbA1c levels and caregiver burden further highlights the interconnectedness of patient clinical outcomes and caregiver well-being, though this relationship may reflect bidirectional pathways rather than a direct causal effect. Future research should further explore the cultural paradox whereby caregiving imposes a burden whilst simultaneously providing satisfaction, in order to develop culturally sensitive interventions.

These findings suggest that clinical practice should consider routine psychosocial screening for family caregivers, particularly when the patient has HbA1c >9.0% or established diabetic complications. Brief validated instruments such as the Zarit Burden Interview-4 (ZBI-4) or the DASS-21 could be used by diabetes nurses or primary care physicians to identify caregivers at elevated risk of burden and psychological distress. When screening is positive, appropriate support, including psychoeducation, referral to social services, or caregiver-focused interventions, should be offered as part of integrated diabetes care.

Future longitudinal studies are needed to establish causal pathways and evaluate the effectiveness of such targeted screening and support programmes. Additionally, the observed attenuation of gender effects from Step 1 to the final model warrants further investigation through formal mediation analysis to clarify potential indirect pathways.

## Data Availability

The datasets generated and/or analyzed during the current study are available from the corresponding author upon reasonable request.
