# Supplementary material for: Care burden and psychological factors in family caregivers of diabetic patients
Source: Rev Assoc Med Bras (1992). 2026 Jul 31;72(6):e20260104. doi: 10.1590/1806-9282.20260104 (PMC13427252; doi:10.1590/1806-9282.20260104)
Supplement: Supplementary Figure 1 [file 1806-9282-ramb-72-06-e20260104-supp1.docx]

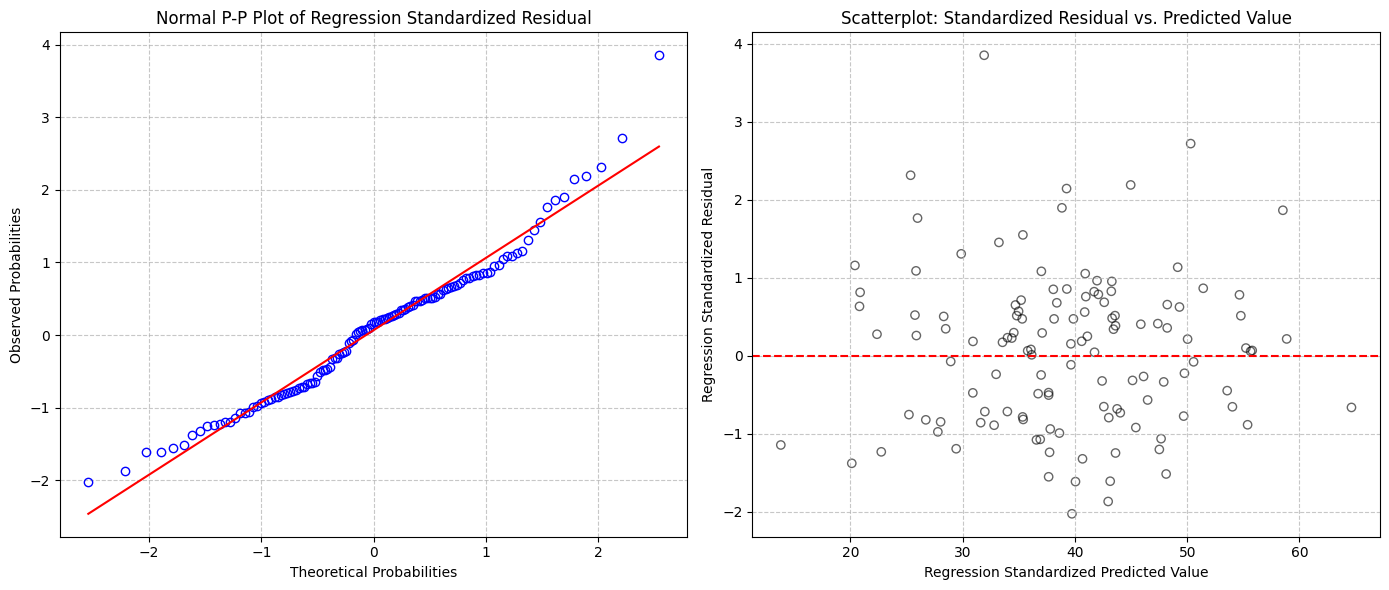


**Supplementary Figure 1.** Residual diagnostics for the final regression model. Left: Normal P–P plot of regression standardized residuals indicates that the error terms follow a normal distribution. Right: Scatterplot of standardized residuals against predicted values shows a random distribution without any discernible pattern (e.g., funneling), confirming the assumption of homoscedasticity. These diagnostics validate the robustness of the hierarchical regression model reported in Table 4.

### **Supplementary Table 1.** Reliability and descriptive statistics of Depression Anxiety Stress Scale-21 subscales (n=125).

| **Subscale** | **Number of ıtems** | **Cronbach’s α** | **Mean±SD** |
| --- | --- | --- | --- |
| Depression | 7 | 0.86 | 8.4±4.1 |
| Anxiety | 7 | 0.84 | 7.6±3.8 |
| Stress | 7 | 0.88 | 9.2±4.5 |
| Total DASS-21 | 21 | 0.92 | 25.2 ± 11.2 |

Note: All Cronbach's α values >0.80 indicate excellent internal consistency. SD: standard deviation; DASS-21: Depression Anxiety Stress Scale-21.

**Supplementary Table 2.** Correlation comparisons by caregiver relationship.

| **Variable pair** | **Spousal caregivers (n=48)** | **Non-spousal caregivers (n=77)** | **p-value (group difference)** |
| --- | --- | --- | --- |
| HbA1c & ZBI score | r=0.70 | r=0.64 | p=0.412 |
| Depression & ZBI score | r=0.49 | r=0.46 | p=0.654 |
| Stress & ZBI score | r=0.53 | r=0.48 | p=0.521 |

Pearson correlation coefficients are presented for each subgroup. All correlations were statistically significant (p<0.001 within groups). HbA1c: glycated hemoglobin; ZBI: Zarit Caregiver Burden Index.

**Supplementary Table 3.** Model diagnostics for the final hierarchical regression model (n=125).

| **Predictor** | **Tolerance** | **VIF** |
| --- | --- | --- |
| Age | 0.88 | 1.14 |
| Gender | 0.82 | 1.22 |
| Relationship (spouse) | 0.75 | 1.33 |
| Income | 0.91 | 1.10 |
| Complications | 0.72 | 1.39 |
| HbA1c | 0.65 | 1.54 |
| Depression | 0.45 | 2.22 |
| Anxiety | 0.48 | 2.08 |
| Stress | 0.42 | 2.38 |

Model-level diagnostics:

- Durbin–Watson=1.92
- Maximum Cook’s distance=0.082

Note: All variance inflation factor (VIF) values were below 2.5, indicating no evidence of problematic multicollinearity. The Durbin–Watson statistic close to 2 suggests independence of residuals. Cook’s distance values were well below 1, indicating no influential outliers. HbA1c: glycated hemoglobin; VIF: variance inflation factor.

**Supplementary Table 4.** Sensitivity analysis: Pearson vs. Spearman correlations.

| **Variable pair** | **Pearson’s r** | **Spearman’s rho** | **p-value** |
| --- | --- | --- | --- |
| HbA1c & ZBI score | 0.684 | 0.672 | <0.001 |
| HbA1c & depression | 0.592 | 0.585 | <0.001 |
| ZBI & stress | 0.510 | 0.495 | <0.001 |

Note: The close agreement between Pearson and Spearman coefficients indicates that the observed associations are robust and not driven by outliers or non-normality. HbA1c: glycated hemoglobin; ZBI: Zarit Caregiver Burden Index.

**Supplementary Table 5.** Breakdown of caregivers exceeding severity thresholds (n=125).

| **Scale** | **Normal level n (%)** | **Elevated level* n (%)** | **Total** |
| --- | --- | --- | --- |
| Depression | 58 (46.4%) | 67 (53.6%) | 125 (100%) |
| Anxiety | 54 (43.2%) | 71 (56.8%) | 125 (100%) |
| Stress | 66 (52.8%) | 59 (47.2%) | 125 (100%) |

Note: “Elevated level” refers to symptoms at mild severity or higher according to Lovibond and Lovibond^11^ criteria.

### **Supplementary Table 6.** Correlation matrix of primary study variables and Depression Anxiety Stress Scale-21 subscales (n=125).

| **Variable** | **1. ZBI score** | **2. Depression** | **3. Anxiety** | **4. Stress** | **5. HbA1c (%)** |
| --- | --- | --- | --- | --- | --- |
| 1. ZBI Score | 1.00 |  |  |  |  |
| 2. Depression | 0.48* | 1.00 |  |  |  |
| 3. Anxiety | 0.42* | 0.64* | 1.00 |  |  |
| 4. Stress | 0.51* | 0.72* | 0.68* | 1.00 |  |
| 5. HbA1c (%) | 0.68* | 0.59* | 0.44* | 0.49* | 1.00 |

*p<0.001. HbA1c: glycated hemoglobin; ZBI: Zarit Caregiver Burden Index.
